# Development of a Rat Model of Intra-Amniotic Inflammation *via* Ultrasound-Guided Administration of a Triggering Agent in the Gestational Sac to Enable Analysis of Individual Amniotic Fluid Samples

**DOI:** 10.3389/fphar.2022.871193

**Published:** 2022-04-12

**Authors:** Jaroslav Stranik, Marian Kacerovsky, Martin Sterba, Ctirad Andrys, Cilia Abad, Frantisek Staud, Stanislav Micuda, Ondrej Soucek, Bo Jacobsson, Ivana Musilova

**Affiliations:** ^1^ Department of Obstetrics and Gynecology, University Hospital Hradec Kralove, Faculty of Medicine in Hradec Kralove, Charles University, Hradec Kralove, Czechia; ^2^ Biomedical Research Center, University Hospital Hradec Kralove, Hradec Kralove, Czechia; ^3^ Department of Pharmacology, Faculty of Medicine in Hradec Kralove, Charles University, Hradec Kralove, Czechia; ^4^ Institute of Clinical Immunology and Allergy, University Hospital Hradec Kralove, Faculty of Medicine in Hradec Kralove, Charles University, Hradec Kralove, Czechia; ^5^ Department of Pharmacology and Toxicology, Faculty of Pharmacy in Hradec Kralove, Charles University, Hradec Kralove, Czechia; ^6^ Department of Obstetrics and Gynecology, Institute of Clinical Science, Sahlgrenska Academy, University of Gothenburg, Gothenburg, Sweden; ^7^ Department of Obstetrics and Gynecology, Region Västra Götaland, Sahlgrenska University Hospital, Gothenburg, Sweden; ^8^ Department of Genetics and Bioinformatics, Domain of Health Data and Digitalization, Institute of Public Health, Oslo, Norway

**Keywords:** animal model, preterm birth, preterm delivery, lipopolysaccharide, minimally invasive, amniocentesis

## Abstract

**Objectives:** To develop a rat model of intra-amniotic inflammation, characterized by the concentration of interleukin-6 in the amniotic fluid, induced by an ultrasound-guided transabdominal administration of lipopolysaccharide into individual gestational sacs.

**Methods:** An ultrasound-guided transabdominal intra-amniotic administration of lipopolysaccharide or phosphate-buffered saline (PBS) as control was performed in rats on embryonic day 18. Only accessible gestational sacs with precise recording of their positions were injected. Twenty-four hours later, individual amniotic fluid samples were collected from the gestational sacs of laparotomized animals. The gestational sacs were divided into four subgroups: (i) with lipopolysaccharide: injected gestational sacs from rats undergoing lipopolysaccharide administration; (ii) without lipopolysaccharide: non-injected gestational sacs from rats undergoing lipopolysaccharide administration; (iii) with PBS: injected gestational sacs from rats undergoing PBS administration; and (iv) without PBS: non-injected gestational sacs from rats undergoing PBS administration. The concentration of interleukin-6 in individual amniotic fluid samples was assessed using ELISA.

**Results:** In the group of five animals receiving lipopolysaccharide, 24 (33%) and 48 (77%) gestational sacs were and were not injected, respectively. The amniotic fluid was obtained from 21 (88%) injected and 46 (95%) non-injected sacs. In the control group of five animals receiving phosphate-buffered saline, 28 (35%) and 52 (75%) gestational sacs were and were not injected, respectively. The amniotic fluid was obtained from 18 (64%) injected and 50 (96%) non-injected sacs. No labor occurred, and only one fetal death was observed in a gestational sac injected with lipopolysaccharide. Differences in concentrations of interleukin-6 in the amniotic fluid were found among the subgroups of the gestational sacs (with lipopolysaccharide: median 762 pg/ml; without lipopolysaccharide: median 35.6 pg/ml; with PBS: median 35.6 pg/ml; and without PBS: median 35.6 pg/ml; *p* < 0.0001). Concentrations of interleukin-6 in the amniotic fluid from the gestational sacs with lipopolysaccharide were significantly higher than those in the three remaining subgroups (*p* < 0.0001). No differences in concentrations of interleukin-6 in the amniotic fluid were identified between the three remaining subgroups.

**Conclusion:** The ultrasound-guided transabdominal intra-amniotic administration of lipopolysaccharide with a subsequent collection and analysis of amniotic fluid samples is feasible in rats. The intra-amniotic administration of lipopolysaccharide led to the development of intra-amniotic inflammation without leading to fetal mortality or induction of labor.

## Introduction

Spontaneous preterm delivery accounts for approximately 8% of all live births worldwide and is the leading cause of perinatal mortality and morbidity (Blencowe et al., 2012; Walani, 2020). It represents one of the “great obstetrical syndromes,” resulting from a multifactorial etiology and complex pathogenesis (Romero et al., 2014a). Intra-amniotic inflammation plays a crucial role in the pathogenesis of preterm delivery and is characterized by the elevation in various inflammatory mediators in the amniotic fluid (Romero et al., 2007). This intra-amniotic complication may be identified in up to 40% of pregnancies with spontaneous preterm delivery (Romero et al., 2014c; Musilova et al., 2015; Kacerovsky et al., 2021; Stranik et al., 2021). Based on the triggering stimulus, two different clinical phenotypes of intra-amniotic inflammation can be distinguished: (i) intra-amniotic infection and (ii) sterile intra-amniotic inflammation when microorganisms and/or their nucleic acids are present or absent in the amniotic fluid, respectively (Romero et al., 2014c; Stranik et al., 2021). Regardless of the nature of intra-amniotic inflammation, its presence remains a serious clinical issue because of its association with adverse pregnancy and neonatal outcomes (Soucy-Giguere et al., 2018).

Animal models represent an important tool in the research on intra-amniotic inflammatory complications in spontaneous preterm births (Nielsen et al., 2016). Compared with human studies, they provide an opportunity for a broad range of study designs, enabling deep and comprehensive insights into the pathogenesis of intra-amniotic inflammatory complications and their association with spontaneous preterm delivery (Spencer et al., 2021). The development of an animal model of intra-amniotic inflammation provides various routes for the administration of triggering agents leading to an intra-amniotic inflammatory response (Elovitz and Mrinalini, 2004). The possible routes of application involve two main approaches: (i) systemic, mainly intraperitoneal application, which is far from a real clinical scenario, and (ii) localized, including vaginal, intracervical, intrauterine, and intra- or extra-amniotic routes (Elovitz and Mrinalini, 2004; Stranik et al., 2020). The intra-amniotic administration of a triggering agent is an ideal approach for the development of a well-defined, local, and intra-amniotic inflammatory response, with the opportunity to study intra-amniotic inflammatory complications and precisely mimic different specific clinical scenarios.

Rodents, particularly mice and rats, are the most frequently used experimental animals to study spontaneous preterm delivery and intra-amniotic inflammatory complications (Nielsen et al., 2016). In these small animals, the administration of triggering agents *via* the intra-amniotic route requires an invasive approach, i.e., laparotomy to access the uterus (Rounioja et al., 2003; Gisslen et al., 2019; Stranik et al., 2020). However, the surgical nature of such an approach is associated with additional stressful stimuli for the animals, particularly for control groups, which might affect the results of the experiment (Rinaldi et al., 2015). To reduce these adverse effects and potential bias, a non-invasive ultrasound-guided transabdominal intra-amniotic administration of triggering agents was introduced by Gomez–Lopez et al. in 2016 for mice (Gomez-Lopez et al., 2016). Unfortunately, the limited amount of amniotic fluid in the gestational sac of mice leads to the use of pooled amniotic fluid from various gestational sacs and prevents the use of individual amniotic fluid samples from a particular gestational sac (Garcia-Flores et al., 2018; Brown et al., 2019; Motomura et al., 2020; Shynlova et al., 2021). This shortcoming can be overcome by using larger animals, such as rats, which have higher volumes of amniotic fluid. In addition, rats are more resistant to labor induction following the administration of an inflammatory agent into the gestational sac (Cookson et al., 2018; Dedja et al., 2018). This prerequisite makes this animal ideal to thoroughly study the intrauterine effects and consequences of intra-amniotic inflammatory complications on the fetus, placenta, and fetal membranes. Collectively, these results suggest that rats are an optimal animal model of intra-amniotic inflammatory complications. However, a rat model based on a non-invasive ultrasound-guided transabdominal intra-amniotic administration of a triggering agent has yet to be developed.

Therefore, the main aim of this study was to establish a rat model of intra-amniotic inflammation, characterized by the concentration of interleukin (IL)-6 in the amniotic fluid, induced by an ultrasound-guided transabdominal administration of *Escherichia coli* (*E. coli*) lipopolysaccharide (LPS) into individual gestational sacs.

## Materials and Methods

### Animals

Pregnant Wistar rats were purchased from Velaz (Prague, Czechia) and housed in the vivarium of the Faculty of Medicine at Hradec Kralove under standard conditions (12 h light/dark cycles, a steady temperature of 22 ± 2°C, a relative air humidity of 50 ± 10%, and water and pellets ad libitum). Embryonic day (E) 1 was defined as the morning when vaginal plug formation occurred. All procedures were performed in accordance with the Act on the Protection of Animals against Cruelty, Act No. 246/1992 Coll., with the approval of the Animal Welfare Committee of the Faculty of Medicine in Hradec Kralove, Charles University and Czech Ministry of Education, Youth and Sports (No. 41058/2016-MZE-17214).

### Ultrasound-Guided Intra-Amniotic Administration of LPS

The intra-amniotic administration was performed on E18. Dams were sedated by the inhalation of 5% isoflurane (Isoflurin, Vetpharma AH, S.L., Barcelona, Spain) with oxygen at 2 L/min in the induction chamber. Anesthesia was maintained with 1.5–2.0% isoflurane and oxygen at 2 L/min. The animals were positioned and fixed on a heating pad from the Vevo Imaging Station (FUJIFILM VisualSonics Inc., Toronto, ON, Canada). The body temperature was maintained at 37 ± 1°C and measured using a rectal thermometer (FUJIFILM VisualSonics Inc., Toronto, ON, Canada). The heart rate and respiratory rate were monitored using electrodes embedded in the heating pad. Fur was removed using a depilatory cream. The ultrasound transducer MX400 (Vevo 3100; FUJIFILM VisualSonics Inc., Toronto, ON, Canada) was placed in a mechanical holder and stabilized at a position that displayed the target gestational sac.

**FIGURE 1:**
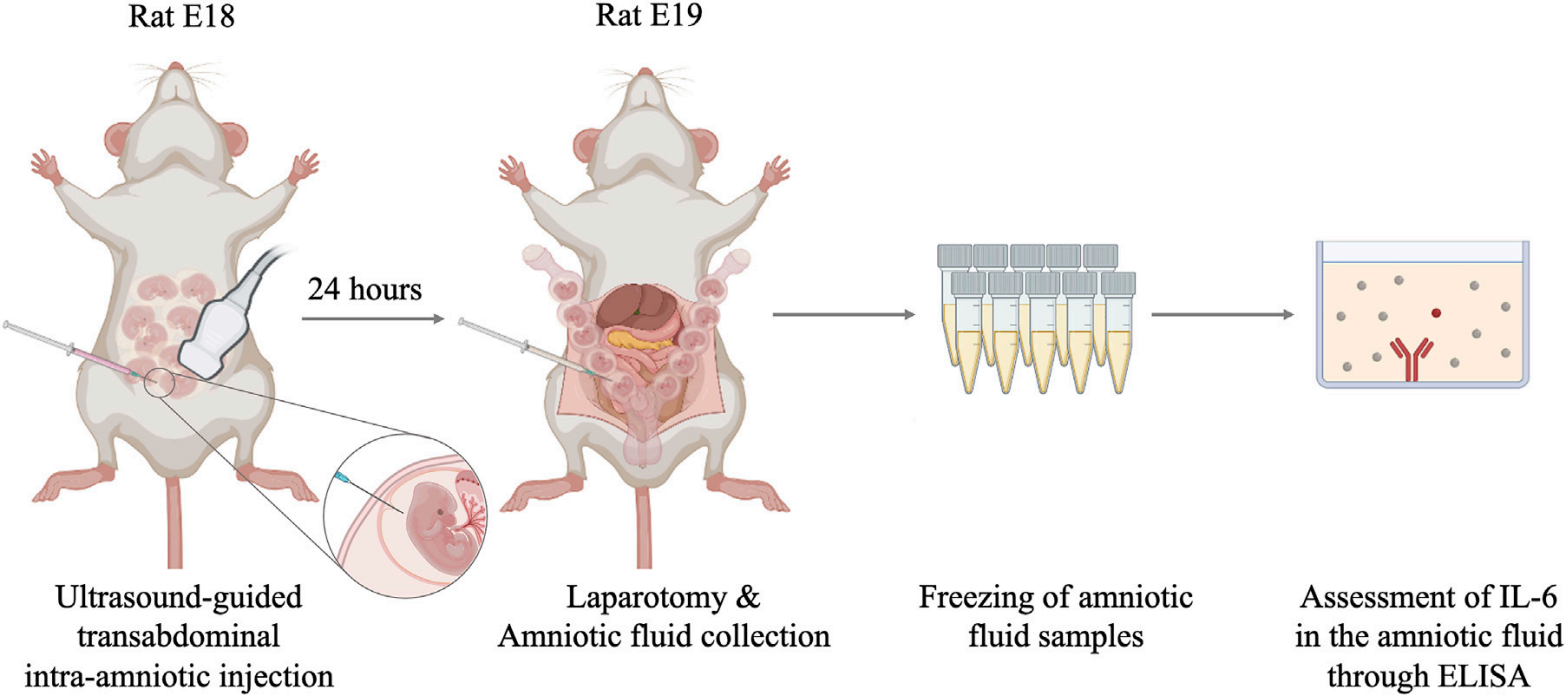
Graphic illustration of the experimental design. IL: interleukin.

Under ultrasound guidance, the intra-amniotic administration of 10 µg of *E. coli* LPS (serotype O55:B5, Sigma-Aldrich, Prague, Czechia) in 100 µL of phosphate-buffered saline (PBS) was performed using a 27 G × 40 mm needle (B. Braun Melsungen, Germany) with an Omnican^®^ 50 syringe (B. Braun, Melsungen, Germany) stabilized in a mechanical holder. The control animals were injected with 100 µL of PBS. Only the accessible gestational sacs with precise recordings of their positions were manipulated (Figure 1). Following the procedure, the animals were kept under a heat lamp for recovery and then returned to their cages.

The gestational sacs were divided into four subgroups based on selective intra-amniotic administration: (i) **gestational sacs with LPS:** injected gestational sacs from rats undergoing LPS administration; (ii) **gestational sacs without LPS:** non-injected gestational sacs from rats undergoing LPS administration; (iii) **gestational sacs with PBS:** injected gestational sacs from rats undergoing PBS administration; and (iv) **gestational sacs without PBS:** non-injected gestational sacs from rats undergoing PBS administration.

### Amniotic Fluid Collection

Twenty-four hours after the intra-amniotic administration, on E19, the animals were anesthetized and prepared for an ultrasound examination in the same manner as for the intra-amniotic administration. The position of the gestational sac and vitality of the pups were assessed using the ultrasound transducer MX250S (15–30 MHz). The uterine horns were exposed using a midline abdominal incision. Before any manipulation of the uterine horns, the injected sacs were identified with respect to the localization recorded a day before administration. After identification, both the uterine horns were removed from the abdominal cavity. Using a sterile 30 G × 13 mm needle (B. Braun Melsungen, Germany), the amniotic fluid was aspirated from all sacs and stored in polypropylene tubes at –70°C until analysis (Figure 1). The placentas, membranes, and fetal tissues were harvested, snap-frozen in liquid nitrogen, and stored at –70°C for further analyses. The animals were sacrificed *via* exsanguination under anesthesia.

### Assessment of Amniotic Fluid IL-6

Concentrations of IL-6 in the amniotic fluid samples were assessed using the Rat IL-6 Quantikine ELISA Kit (R&D Systems Inc., Minneapolis, MN, United States) according to the manufacturer’s instructions. The sensitivity of the kit was 36 pg/ml, and the inter-assay and intra-assay coefficients were <9% and <10%, respectively. The absorbance was measured at 450 nm using a Multiskan RC ELISA reader (Thermo Fisher Scientific, Waltham, MA, United States).

### Statistical Analyses

The normality of the data was tested using the Anderson–Darling test. The concentrations of IL-6 in the amniotic fluid were not normally distributed, therefore, nonparametric Kruskal–Wallis or Mann–Whitney *U* tests were used for analyses. All *p* values were determined using two-tailed tests, and all statistical analyses were performed using GraphPad Prism v8 for Mac OS X (GraphPad Software, San Diego, CA, United States).

## Results

### Animal Characteristics

**FIGURE 2:**
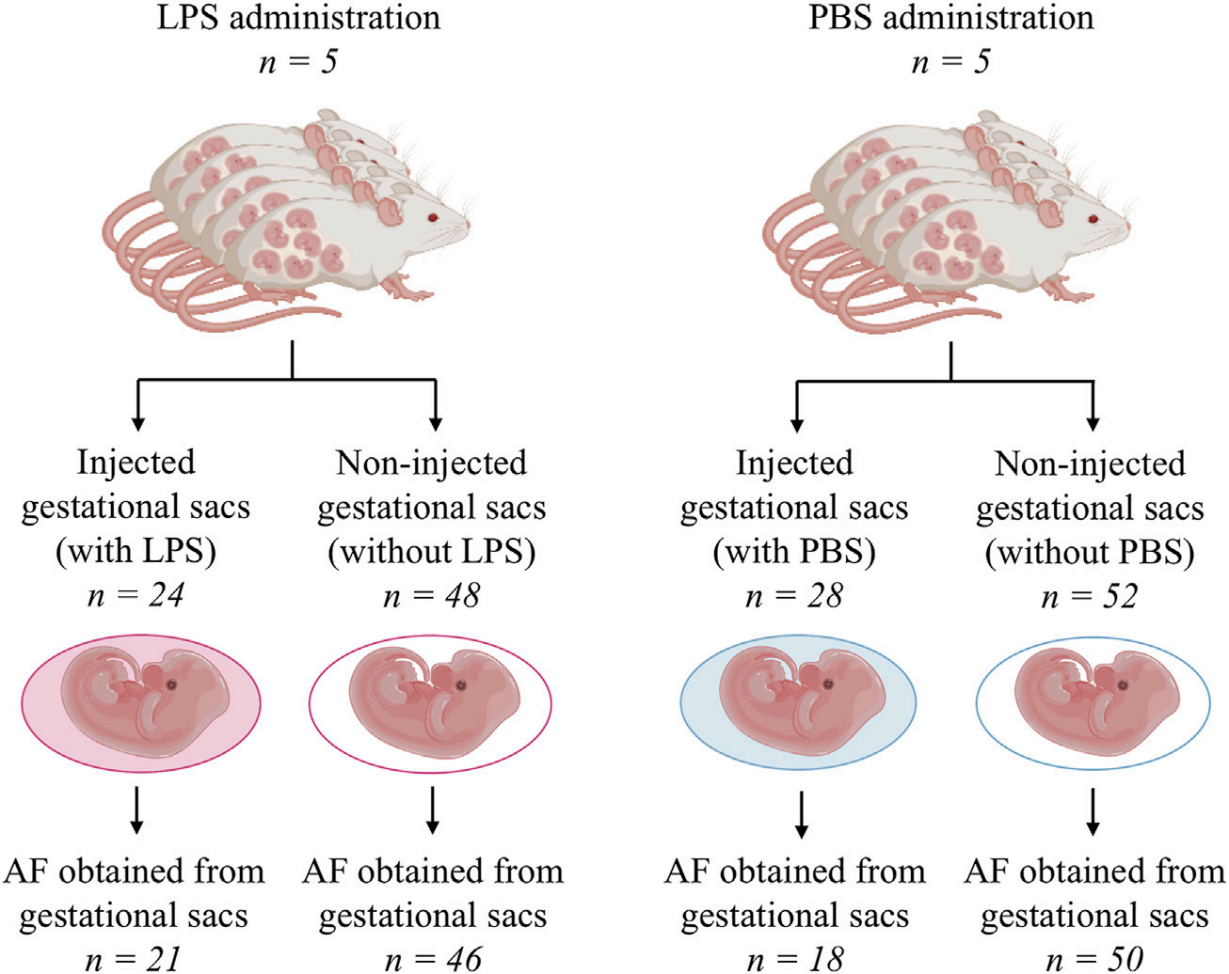
Schema of the flow through the animal experiment. AF, amniotic fluid; LPS, lipopolysaccharide; and PBS, phosphate-buffered saline.

In total, ten rats were included in the study, of which five received LPS and five were administered PBS only. In the group of animals receiving LPS (n = 5), from a total number of 72 gestational sacs, 24 (33%) and 48 (77%) were and were not injected with LPS, respectively. In the group of animals receiving PBS only, from a total number of 80 gestational sacs, 28 (35%) and 52 (75%) were and were not injected with PBS, respectively (Figure 2).

### Parturition Initiation

Labor did not occur in any dam within 24 h following administration.

### Fetal Mortality

One intrauterine fetal death was observed in the subgroup of the gestational sacs with LPS, and the mortality rate of this subgroup was 4% (1/28). No amniotic fluid was received from the gestational sac owing to anhydramnios. All fetuses from the other subgroups survived.

### Amniotic Fluid Collection

In the group of animals receiving LPS, an amniotic fluid volume sufficient for analysis was obtained from 21 (88%) gestational sacs injected with LPS and from 46 (95%) gestational sacs without LPS (Figure 2). In the PBS group, an amniotic fluid volume sufficient for analysis was obtained from 18 (64%) gestational sacs injected with PBS and 50 (96%) gestational sacs without PBS (Figure 2).

### Concentration of IL-6 in the Amniotic Fluid After Intra-Amniotic LPS Administration

**FIGURE 3:**
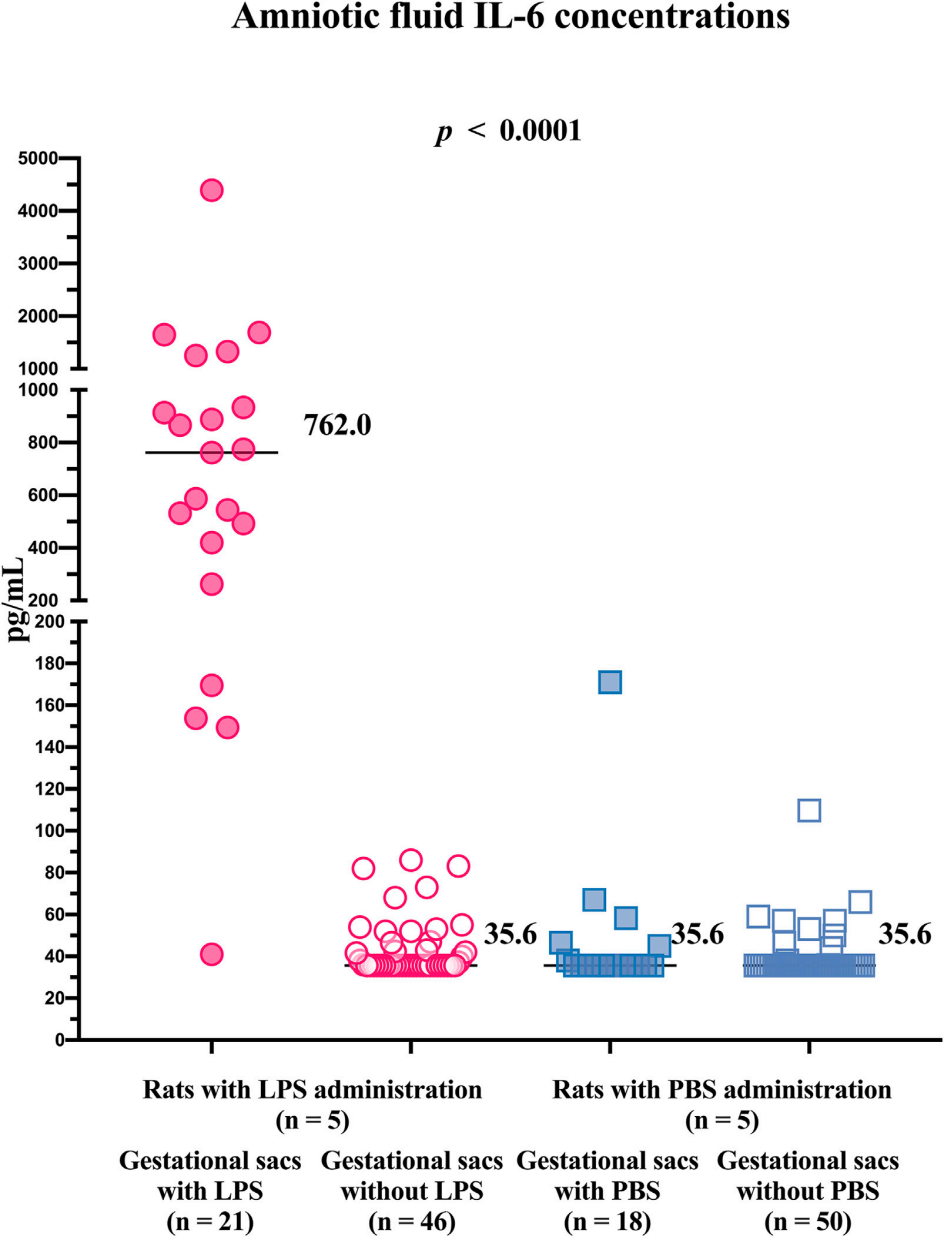
Concentrations of IL-6 in the amniotic fluid from individual gestational sacs of rats after an ultrasound-guided transabdominal intra-amniotic administration of either lipopolysaccharide or phosphate-buffered saline. LPS, lipopolysaccharide; PBS, phosphate-buffered saline; and IL, interleukin.

Differences in the concentration of IL-6 in the amniotic fluid were found among the subgroups of the gestational sacs (gestational sacs with LPS: median 762 pg/ml; IQR 340.8–1,093 pg/ml; gestational sacs without LPS: median 35.6 pg/ml, IQR 35.6–46.63 pg/ml; gestational sacs with PBS: median 35.6 pg/ml, IQR 35.6–45.38 pg/ml; and gestational sacs without PBS: median 35.6 pg/ml, IQR 35.6–35.6 pg/ml; *p* ≤ 0.0001; Figure 3).

The concentrations of IL-6 in the amniotic fluid samples obtained from the gestational sacs with LPS were higher than those in the amniotic fluid samples obtained from gestational sacs without LPS and those with or without PBS (Table 1). No differences in the concentrations of IL-6 in the amniotic fluid were identified between the gestational sacs without LPS and those with or without PBS (Table 1).

**TABLE 1:** Comparisons of interleukin-6 concentrations in the amniotic fluid from individual gestational sacs after an ultrasound-guided transabdominal intra-amniotic administration of either lipopolysaccharide or phosphate-buffered saline in rats.

		Administration of lipopolysaccharide (LPS)		Administration of phosphate-buffered saline (PBS)	
		Gestational sacs with LPS	Gestational sacs without LPS	Gestational sacs with PBS	Gestational sacs without PBS
**Administration of LPS**	Gestational sacs with LPS	x	***p* < 0.0001**	***p* < 0.0001**	***p* < 0.0001**
	Gestational sac without LPS	***p* < 0.0001**	x	*p* = 1.00	*p* = 0.61
**Administration of PBS**	Gestational sac with PBS	***p* < 0.0001**	*p* = 1.00	x	*p* = 1.00
	Gestational sac without PBS	***p* < 0.0001**	*p* = 0.61	*p* = 1.00	x

Comparisons were performed using a nonparametric Mann–Whitney U test.

Statistically significant results are marked in bold.

## Discussion

The principal findings of this study were as follows: i) the ultrasound-guided transabdominal intra-amniotic administration of an agent was a feasible procedure in rats, ii) the ultrasound-guided transabdominal intra-amniotic administration of 10 µg of *E. coli* LPS serotype O55:B5 did not induce labor within 24 h after administration in rats, iii) fetal mortality associated with the ultrasound-guided transabdominal intra-amniotic administration of 10 µg of *E. coli* LPS serotype O55:B5 was at 4%, iv) the collection of individual amniotic fluid samples from the gestational sacs was feasible, v) the ultrasound-guided transabdominal intra-amniotic administration of 10 µg of *E. coli* LPS serotype O55:B5 in rats led to elevated concentrations of IL-6 in the amniotic fluid only in the injected gestational sacs, and vi) there were no elevations in IL-6 concentrations in the amniotic fluid of gestational sacs not injected with LPS.

In small laboratory animals, the administration of a triggering agent under ultrasound guidance has become possible owing to the advent of high-frequency ultrasound devices designed specifically for small animals (Greco et al., 2013; Galaz et al., 2020). Ultrasound-guided procedures have been used to induce intra-amniotic inflammation in mice (Gomez-Lopez et al., 2016; Gomez-Lopez et al., 2018; Faro et al., 2019; Gomez-Lopez et al., 2019; Motomura et al., 2020). The first use of ultrasound guidance for transabdominal injections was reported by Rinaldi et al. (2015), but this approach was used for intrauterine extra-amniotic administration. Ultrasound-guided transabdominal intra-amniotic administration in mice has become the standard method for the research group of Gomez–Lopez (Gomez-Lopez et al., 2016; Gomez-Lopez et al., 2016; Gomez-Lopez et al., 2018; Faro et al., 2019; Gomez-Lopez et al., 2019; Motomura et al., 2020). The present absence of the use of an ultrasound-guided transabdominal intra-amniotic administration of a triggering agent in rats may be owing to multiple reasons, nevertheless, the thicker rat skin impeding needle passage through the abdominal wall might play a substantive role. This obstacle might be resolved by slowly puncturing the skin to allow the tip of the needle to spontaneously slide through the skin, abdominal and uterine walls, and membranes of the gestational sac.

There is evidence that the intra-amniotic administration of LPS to mice causes preterm delivery in most animals (Garcia-Flores et al., 2018; Gomez-Lopez et al., 2018; Faro et al., 2019). In our study, the dams did not deliver within 24 h after the intra-amniotic administration of LPS despite the development of an intra-amniotic inflammation, characterized by the elevation in IL-6 concentrations in the amniotic fluid. This was in line with other rat studies, in which the intra-amniotic administration of LPS via laparotomy did not induce delivery (Cookson et al., 2018; Dedja et al., 2018). The fact that the rat animal model of intra-amniotic inflammation was not associated with the risk of labor induction within 24 h is important from a research standpoint because it provides an opportunity to collect various body fluids and tissues from gestational sacs after their exposure to intra-amniotic inflammation. This approach mimics human clinical scenarios (Musilova et al., 2018; Kacerovsky et al., 2020b).

The preservation of the vitality of the fetus after a triggering agent administration represents an important key feature of the animal model of local intra-amniotic inflammation, mimicking the clinical scenario of human pregnancy complicated by intra-amniotic inflammation (Musilova et al., 2015). The presence of a vital fetus allows the possibility of studying the consequences of intra-amniotic inflammation on the fetus *in utero*, for example, via ultrasonography or other diagnostic methods. The intra-amniotic administration of LPS in mice models is related to a significantly high mortality rate, however, such a phenomenon has not been reported in rat studies (Cookson et al., 2018; Gomez-Lopez et al., 2018; Jantzie et al., 2018; Faro et al., 2019). Fetal mortality in mice due to LPS administration is dependent on the dose and type of LPS (Migale et al., 2015), however, based on the rare occurrence of fetal mortality after the intra-amniotic administration of LPS in rats, this animal model seems to be less sensitive to this complication. These observations are in agreement with those of our study, where only one intrauterine fetal death occurred among 28 fetuses from the gestational sacs with intra-amniotic inflammation (injected with LPS). This intrauterine demise might be considered a direct consequence of intra-amniotic inflammation, however, the fetal trauma associated with the gestational sac puncture, as a cause of death, cannot be completely excluded.

In human clinical practice, intra-amniotic inflammation is identified based on the assessment of various inflammatory markers (such as IL-6, matrix metalloproteinase 8, or glucose) in the sampled amniotic fluid (Nien et al., 2006; Kacerovsky et al., 2014; Kacerovsky et al., 2020a; Oh et al., 2020). Therefore, it is of utmost relevance to have individual amniotic fluid samples available from animal models to study changes in amniotic fluid composition under various research scenarios. Thus far, the analysis of pooled samples has been preferred owing to the low volume of the amniotic fluid obtained from the gestational sacs in small laboratory animals (Awad et al., 2011; Garcia-Flores et al., 2018; Simoes et al., 2018; Motomura et al., 2020). In this study, individual amniotic fluid samples from rats were analyzed. The amount of amniotic fluid obtained from gestational sacs was sufficient to assess IL-6 using a commercially available ELISA kit in a standard manner. Regardless of the limited amount of amniotic fluid obtained from each gestational sac, we believe that this amount would be sufficient for running most antibody-based assays, however, it might restrict the spectrum or number of analytes that can be assessed. It is worth mentioning that intra-amniotic administration/injection is prone to the reduction of the amniotic fluid volume owing to i) a possible leakage of the amniotic fluid from the gestational sac through the site of fetal membrane perforation and ii) an alteration of the amniotic fluid production by the fetal membranes owing to the development of an intra-amniotic inflammatory response.

LPS, a component of the cell wall of Gram-negative bacteria, has been a typical triggering agent that has been inducing inflammation in animal models for decades (Kemp et al., 2010). In clinical scenarios, Gram-negative bacteria are not the most common microorganisms causing intra-amniotic complications (Musilova et al., 2015; Musilova et al., 2017). However, the strong potential of LPS to trigger a severe intra-amniotic inflammatory response leading to the development of intra-amniotic inflammation was the reason for using this triggering agent in the development of this animal model. Systemic LPS administration to pregnant rats was followed by the production of IL-6, IL-1β, and the tumor necrosis factor-α in the amniotic fluid (Urakubo et al., 2001; Beloosesky et al., 2006; Awad et al., 2011). However, no studies have assessed the levels of inflammatory mediators in the amniotic fluid following intra-amniotic administration in rats. In our study, the intra-amniotic injection of 10 µg of *E. coli* LPS serotype O55:B5 per gestational sac triggered a marked elevation in the IL-6 concentration in the amniotic fluid. This elevation was observed only in gestational sacs injected with LPS, whereas no changes in the concentrations of IL-6 were identified in the non-injected gestational sacs from the same dam 24 h after LPS administration. The concurrent presence of injected and non-injected gestational sacs in one uterus offers a scenario clinically relevant to multiple pregnancies, as the presence of intra-amniotic inflammation associated with spontaneous preterm delivery in twins may be observed in only one of them (Oh et al., 2019).

This study has several strengths. First, a minimally invasive approach for the intra-amniotic administration of LPS was used. Second, the intensity of the intra-amniotic inflammatory response in individual gestational sacs was determined *via* the analysis of selected inflammatory mediators in the amniotic fluid. The individual analysis of amniotic fluid samples after the selective intra-amniotic administration of various triggering agents provides the opportunity to study differences in the intensities of intra-amniotic inflammatory responses not only between various dams but also between injected and non-injected gestational sacs originating from one dam.

Nevertheless, the study has some limitations. First, only one dose of LPS was used. We used an already proven dose of 10 μg *E. coli* LPS serotype O55:B5 that induces fetal lung inflammatory injury and does not lead to preterm delivery (Cookson et al., 2018). Second, only one 24-h interval from LPS administration to amniotic fluid sampling was used. The absence of other time intervals did not allow us to describe the temporal relationship between intra-amniotic administration of triggering agents and the development of intra-amniotic inflammatory response. Third, only one thoroughly selected inflammatory mediator was used to determine the intensity of intra-amniotic inflammation. Nevertheless, IL-6 is considered the gold standard marker in the identification of intra-amniotic inflammation, superior to classical markers such as glucose, lactate, and white blood cell counts and is not inferior to modern proteomic markers (Romero et al., 1993a; Romero et al., 1993b; Romero et al., 2014b).

In conclusion, the ultrasound-guided transabdominal intra-amniotic administration of LPS with subsequent collection and analysis of amniotic fluid samples was feasible in rats. The intra-amniotic administration of LPS led to the development of intra-amniotic inflammation without fetal mortality or induction of labor.

## Data Availability

The raw data supporting the conclusion of this article will be made available by the authors, without undue reservation.

## References

[B1] Awad N. Khatib N. Ginsberg Y. Weiner Z. Maravi N. Thaler I. (2011). N-acetyl-cysteine (NAC) Attenuates LPS-Induced Maternal and Amniotic Fluid Oxidative Stress and Inflammatory Responses in the Preterm Gestation. Am. J. Obstet. Gynecol. 204, 450.e15-20. 10.1016/j.ajog.2011.01.030 21411055

[B2] Beloosesky R. Gayle D. A. Amidi F. Nunez S. E. Babu J. Desai M. (2006). N-acetyl-cysteine Suppresses Amniotic Fluid and Placenta Inflammatory Cytokine Responses to Lipopolysaccharide in Rats. Am. J. Obstet. Gynecol. 194, 268–273. 10.1016/j.ajog.2005.06.082 16389042

[B3] Blencowe H. Cousens S. Oestergaard M. Z. Chou D. Moller A. B. Narwal R. (2012). National, Regional, and Worldwide Estimates of Preterm Birth Rates in the Year 2010 with Time Trends since 1990 for Selected Countries: a Systematic Analysis and Implications. Lancet 379, 2162–2172. 10.1016/S0140-6736(12)60820-4 22682464

[B4] Brown A. G. Maubert M. E. Anton L. Heiser L. M. Elovitz M. A. (2019). The Tracking of Lipopolysaccharide through the Feto-Maternal Compartment and the Involvement of Maternal TLR4 in Inflammation-Induced Fetal Brain Injury. Am. J. Reprod. Immunol. 82, e13189. 10.1111/aji.13189 31495009 PMC6899932

[B5] Cookson M. W. Ryan S. L. Seedorf G. J. Dodson R. B. Abman S. H. Mandell E. W. (2018). Antenatal Vitamin D Preserves Placental Vascular and Fetal Growth in Experimental Chorioamnionitis Due to Intra-amniotic Endotoxin Exposure. Am. J. Perinatol 35, 1260–1270. 10.1055/s-0038-1642033 29715698

[B6] Dedja A. Gucciardi A. Giordano G. Maria Di Gangi I. Porzionato A. Navaglia F. (2018). Lipopolysaccharide-induced Chorioamnionitis and Postnatal Lung Injury: The Beneficial Effects of L-Citrulline in Newborn Rats. Exp. Lung Res. 44, 226–240. 10.1080/01902148.2018.1497730 30198795

[B7] Elovitz M. A. Mrinalini C. (2004). Animal Models of Preterm Birth. Trends Endocrinol. Metab. 15, 479–487. 10.1016/j.tem.2004.10.009 15541647

[B8] Faro J. Romero R. Schwenkel G. Garcia-Flores V. Arenas-Hernandez M. Leng Y. (2019). Intra-amniotic Inflammation Induces Preterm Birth by Activating the NLRP3 Inflammasome†. Biol. Reprod. 100, 1290–1305. 10.1093/biolre/ioy261 30590393 PMC6698670

[B9] Galaz J. Romero R. Arenas-Hernandez M. Panaitescu B. Garcia-Flores V. Gomez-Lopez N. (2020). A Protocol for Evaluating Vital Signs and Maternal-Fetal Parameters Using High-Resolution Ultrasound in Pregnant Mice. STAR Protoc. 1, 100134. 10.1016/j.xpro.2020.100134 33377028 PMC7757336

[B10] Garcia-Flores V. Romero R. Miller D. Xu Y. Done B. Veerapaneni C. (2018). Inflammation-Induced Adverse Pregnancy and Neonatal Outcomes Can Be Improved by the Immunomodulatory Peptide Exendin-4. Front. Immunol. 9, 1291. 10.3389/fimmu.2018.01291 29967606 PMC6015905

[B11] Gisslen T. Singh G. Georgieff M. K. (2019). Fetal Inflammation Is Associated with Persistent Systemic and Hippocampal Inflammation and Dysregulation of Hippocampal Glutamatergic Homeostasis. Pediatr. Res. 85, 703–710. 10.1038/s41390-019-0330-y 30745569 PMC6435426

[B12] Gomez-lopez N. Romero R. Arenas-Hernandez M. Panaitescu B. Garcia-Flores V. Mial T. N. (2018). Intra-amniotic Administration of Lipopolysaccharide Induces Spontaneous Preterm Labor and Birth in the Absence of a Body Temperature Change. J. Matern. Fetal Neonatal. Med. 31, 439–446. 10.1080/14767058.2017.1287894 28139962 PMC5634940

[B13] Gomez-lopez N. Romero R. Garcia-Flores V. Leng Y. Miller D. Hassan S. S. (2019). Inhibition of the NLRP3 Inflammasome Can Prevent Sterile Intra-amniotic Inflammation, Preterm Labor/birth, and Adverse Neonatal Outcomes†. Biol. Reprod. 100, 1306–1318. 10.1093/biolre/ioy264 30596885 PMC6497524

[B14] Gomez-lopez N. Romero R. Plazyo O. Panaitescu B. Furcron A. E. Miller D. (2016). Intra-Amniotic Administration of HMGB1 Induces Spontaneous Preterm Labor and Birth. Am. J. Reprod. Immunol. 75, 3–7. 10.1111/aji.12443 26781934 PMC5029853

[B15] Greco A. Ragucci M. Coda A. R. Rosa A. Gargiulo S. Liuzzi R. (2013). High Frequency Ultrasound for *In Vivo* Pregnancy Diagnosis and Staging of Placental and Fetal Development in Mice. PLoS One 8, e77205. 10.1371/journal.pone.0077205 24155928 PMC3796510

[B16] Jantzie L. L. Oppong A. Y. Conteh F. S. Yellowhair T. R. Kim J. Fink G. (2018). Repetitive Neonatal Erythropoietin and Melatonin Combinatorial Treatment Provides Sustained Repair of Functional Deficits in a Rat Model of Cerebral Palsy. Front. Neurol. 9, 233. 10.3389/fneur.2018.00233 29706928 PMC5908903

[B17] Kacerovsky M. Holeckova M. Stepan M. Gregor M. Vescicik P. Lesko D. (2020a). Amniotic Fluid Glucose Level in PPROM Pregnancies: a Glance at the Old Friend. J. Matern. Fetal Neonatal. Med., 1–13. 10.1080/14767058.2020.1783232 32580603

[B18] Kacerovsky M. Musilova I. Hornychova H. Kutova R. Pliskova L. Kostal M. (2014). Bedside Assessment of Amniotic Fluid Interleukin-6 in Preterm Prelabor Rupture of Membranes. Am. J. Obstet. Gynecol. 211, 385–389. 10.1016/j.ajog.2014.03.069 24705131

[B19] Kacerovsky M. Romero R. Stepan M. Stranik J. Maly J. Pliskova L. (2020b). Antibiotic Administration Reduces the Rate of Intraamniotic Inflammation in Preterm Prelabor Rupture of the Membranes. Am. J. Obstet. Gynecol. 223, 114–e20. 10.1016/j.ajog.2020.01.043 32591087 PMC9125527

[B20] Kacerovsky M. Stranik J. Kukla R. Bolehovska R. Bostik P. Matulova J. (2021). Intra-amniotic Infection and Sterile Intra-amniotic Inflammation in Women with Preterm Labor with Intact Membranes Are Associated with a Higher Rate of Ureaplasma Species DNA Presence in the Cervical Fluid. J. Maternal-Fetal Neonatal Med., 1–9. 10.1080/14767058.2021.1947231 34238107

[B21] Kemp M. W. Saito M. Newnham J. P. Nitsos I. Okamura K. Kallapur S. G. (2010). Preterm Birth, Infection, and Inflammation Advances from the Study of Animal Models. Reprod. Sci. 17, 619–628. 10.1177/1933719110373148 20581349

[B22] Migale R. Herbert B. R. Lee Y. S. Sykes L. Waddington S. N. Peebles D. (2015). Specific Lipopolysaccharide Serotypes Induce Differential Maternal and Neonatal Inflammatory Responses in a Murine Model of Preterm Labor. Am. J. Pathol. 185, 2390–2401. 10.1016/j.ajpath.2015.05.015 26212908 PMC4597270

[B23] Motomura K. Romero R. Xu Y. Theis K. R. Galaz J. Winters A. D. (2020). Intra-Amniotic Infection with Ureaplasma Parvum Causes Preterm Birth and Neonatal Mortality that Are Prevented by Treatment with Clarithromycin. mBio 11. 10.1128/mBio.00797-20 PMC7315120 32576673

[B24] Musilova I. Andrys C. Hornychova H. Pliskova L. Drahosova M. Zednikova B. (2018). Gastric Fluid Used to Assess Changes during the Latency Period in Preterm Prelabor Rupture of Membranes. Pediatr. Res. 84, 240–247. 10.1038/s41390-018-0073-1 29892034

[B25] Musilova I. Kutová R. Pliskova L. Stepan M. Menon R. Jacobsson B. (2015). Intraamniotic Inflammation in Women with Preterm Prelabor Rupture of Membranes. PLoS One 10, e0133929. 10.1371/journal.pone.0133929 26208287 PMC4514652

[B26] Musilova I. Pliskova L. Gerychova R. Janku P. Simetka O. Matlak P. (2017). Maternal white Blood Cell Count Cannot Identify the Presence of Microbial Invasion of the Amniotic Cavity or Intra-amniotic Inflammation in Women with Preterm Prelabor Rupture of Membranes. PLoS One 12, e0189394. 10.1371/journal.pone.0189394 29232399 PMC5726631

[B27] Nielsen B. W. Bonney E. A. Pearce B. D. Donahue L. R. Sarkar I. N., and PRETERM BIRTH INTERNATIONAL (2016). A Cross-Species Analysis of Animal Models for the Investigation of Preterm Birth Mechanisms. Reprod. Sci. 23, 482–491. 10.1177/1933719115604729 26377998 PMC5933186

[B28] Nien J. K. Yoon B. H. Espinoza J. Kusanovic J. P. Erez O. Soto E. (2006). A Rapid MMP-8 Bedside Test for the Detection of Intra-amniotic Inflammation Identifies Patients at Risk for Imminent Preterm Delivery. Am. J. Obstet. Gynecol. 195, 1025–1030. 10.1016/j.ajog.2006.06.054 17000236

[B29] Oh K. J. Hong J. S. Romero R. Yoon B. H. (2019). The Frequency and Clinical Significance of Intra-amniotic Inflammation in Twin Pregnancies with Preterm Labor and Intact Membranes. J. Matern. Fetal Neonatal. Med. 32, 527–541. 10.1080/14767058.2017.1384460 29020827 PMC5899042

[B30] Oh K. J. Lee J. Romero R. Park H. S. Hong J. S. Yoon B. H. (2020). A New Rapid Bedside Test to Diagnose and Monitor Intraamniotic Inflammation in Preterm PROM Using Transcervically Collected Fluid. Am. J. Obstet. Gynecol. 223, 423–e15. 10.1016/j.ajog.2020.02.037 32114081 PMC9521159

[B31] Rinaldi S. F. Makieva S. Frew L. Wade J. Thomson A. J. Moran C. M. (2015). Ultrasound-guided Intrauterine Injection of Lipopolysaccharide as a Novel Model of Preterm Birth in the Mouse. Am. J. Pathol. 185, 1201–1206. 10.1016/j.ajpath.2015.01.009 25747535 PMC4419281

[B32] Romero R. Dey S. K. Fisher S. J. (2014a). Preterm Labor: One Syndrome, many Causes. Science 345, 760–765. 10.1126/science.1251816 25124429 PMC4191866

[B33] Romero R. Gotsch F. Pineles B. Kusanovic J. P. (2007). Inflammation in Pregnancy: its Roles in Reproductive Physiology, Obstetrical Complications, and Fetal Injury. Nutr. Rev. 65, S194–S202. 10.1111/j.1753-4887.2007.tb00362.x 18240548

[B34] Romero R. Kadar N. Miranda J. Korzeniewski S. J. Schwartz A. G. Chaemsaithong P. (2014b). The Diagnostic Performance of the Mass Restricted (MR) Score in the Identification of Microbial Invasion of the Amniotic Cavity or Intra-amniotic Inflammation Is Not superior to Amniotic Fluid Interleukin-6. J. Matern. Fetal Neonatal. Med. 27, 757–769. 10.3109/14767058.2013.844123 24028673 PMC5881917

[B35] Romero R. Miranda J. Chaiworapongsa T. Korzeniewski S. J. Chaemsaithong P. Gotsch F. (2014c). Prevalence and Clinical Significance of Sterile Intra-amniotic Inflammation in Patients with Preterm Labor and Intact Membranes. Am. J. Reprod. Immunol. 72, 458–474. 10.1111/aji.12296 25078709 PMC4192099

[B36] Romero R. Yoon B. H. Mazor M. Gomez R. Diamond M. P. Kenney J. S. (1993a). The Diagnostic and Prognostic Value of Amniotic Fluid white Blood Cell Count, Glucose, Interleukin-6, and Gram Stain in Patients with Preterm Labor and Intact Membranes. Am. J. Obstet. Gynecol. 169, 805–816. 10.1016/0002-9378(93)90009-8 7694461

[B37] Romero R. Yoon B. H. Mazor M. Gomez R. Gonzalez R. Diamond M. P. (1993b). A Comparative Study of the Diagnostic Performance of Amniotic Fluid Glucose, white Blood Cell Count, Interleukin-6, and Gram Stain in the Detection of Microbial Invasion in Patients with Preterm Premature Rupture of Membranes. Am. J. Obstet. Gynecol. 169, 839–851. 10.1016/0002-9378(93)90014-a 7694463

[B38] Rounioja S. Räsänen J. Glumoff V. Ojaniemi M. Mäkikallio K. Hallman M. (2003). Intra-amniotic Lipopolysaccharide Leads to Fetal Cardiac Dysfunction. A Mouse Model for Fetal Inflammatory Response. Cardiovasc. Res. 60, 156–164. 10.1016/s0008-6363(03)00338-9 14522418

[B39] Shynlova O. Nadeem L. Dorogin A. Mesiano S. Lye S. J. (2021). The Selective Progesterone Receptor Modulator-Promegestone-Delays Term Parturition and Prevents Systemic Inflammation-Mediated Preterm Birth in Mice. Am. J. Obstet. Gynecol. 226, 249.e1–249.e21. 34418351 10.1016/j.ajog.2021.08.013 PMC8810746

[B40] Simões L. R. Sangiogo G. Tashiro M. H. Generoso J. S. Faller C. J. Dominguini D. (2018). Maternal Immune Activation Induced by Lipopolysaccharide Triggers Immune Response in Pregnant Mother and Fetus, and Induces Behavioral Impairment in Adult Rats. J. Psychiatr. Res. 100, 71–83. 10.1016/j.jpsychires.2018.02.007 29494891

[B41] Soucy-Giguère L. Gasse C. Giguère Y. Demers S. Bujold E. Boutin A. (2018). Intra-amniotic Inflammation and Child Neurodevelopment: a Systematic Review Protocol. Syst. Rev. 7, 12. 10.1186/s13643-018-0683-z 29357925 PMC5778727

[B42] Spencer N. R. Radnaa E. Baljinnyam T. Kechichian T. Tantengco O. A. G. Bonney E. (2021). Development of a Mouse Model of Ascending Infection and Preterm Birth. PLoS One 16, e0260370. 10.1371/journal.pone.0260370 34855804 PMC8638907

[B43] Stranik J. Kacerovsky M. Soucek O. Kolackova M. Musilova I. Pliskova L. (2021). IgGFc-binding Protein in Pregnancies Complicated by Spontaneous Preterm Delivery: a Retrospective Cohort Study. Sci. Rep. 11, 6107. 10.1038/s41598-021-85473-2 33731725 PMC7969627

[B44] Stranik J. Kacerovsky M. Vescicik P. Faist T. Jacobsson B. Musilova I. (2020). A Rodent Model of Intra-amniotic Inflammation/infection, Induced by the Administration of Inflammatory Agent in a Gestational Sac, Associated with Preterm Delivery: a Systematic Review. J. Matern. Fetal Neonatal. Med. 35, 1–9. 10.1080/14767058.2020.1757063 32349576

[B45] Urakubo A. Jarskog L. F. Lieberman J. A. Gilmore J. H. (2001). Prenatal Exposure to Maternal Infection Alters Cytokine Expression in the Placenta, Amniotic Fluid, and Fetal Brain. Schizophr Res. 47, 27–36. 10.1016/s0920-9964(00)00032-3 11163542

[B46] Walani S. R. (2020). Global burden of Preterm Birth. Int. J. Gynaecol. Obstet. 150, 31–33. 10.1002/ijgo.13195 32524596

